# Professional relationships during crisis interventions: A scoping review

**DOI:** 10.1371/journal.pone.0298726

**Published:** 2024-02-23

**Authors:** Larissa Steimle, Sebastian von Peter, Fabian Frank

**Affiliations:** 1 Department of Psychiatry and Psychotherapy, Brandenburg Medical School Theodor Fontane, Neuruppin, Germany; 2 Faculty of Health and Social Work, Frankfurt University of Applied Sciences, Frankfurt, Germany; 3 Department of Social Work, Protestant University of Applied Sciences Freiburg, Freiburg, Germany; University of Saskatchewan, CANADA

## Abstract

**Introduction:**

A crisis can be described as subjective experience that threatens and overwhelms a person’s ability to handle a specific situation. In dealing with crises some people are looking for support from professionals. The “professional relationship” between people experiencing a crisis and professionals plays an important role in the successful management of a crisis which has been widely researched in many contexts. However, regarding outpatient services (e. g. crisis resolution home treatment teams), yet empirical evidence remains limited.

**Objective:**

We aim to explore descriptions of supportive professional relationships during outpatient crisis interventions in empirical literature. Accordingly, a scoping review was conducted to identify types of evidence, map the key concepts, and point out research gaps.

**Methods:**

MEDLINE, PsycINFO, CINAHL and Social Science Citation Index were searched for studies reporting empirical data on the professional relationship between people experiencing a crisis (18+) and professionals (e. g. social workers, psychiatrists) during a crisis intervention, defined as a short-term, face-to-face, low threshold, time-limited, outpatient, and voluntary intervention to cope with crises. Studies were excluded if they were published before 2007, in languages other than English and German, and if they couldn’t be accessed. Included studies were summarized, compared, and synthesized using qualitative content analyses.

**Results:**

3.741 records were identified, of which 8 met the eligibility criteria. Only one study directly focused on the relationship; the others addressed varied aspects. Two studies explored the perspectives of service users, five focused on those of the professionals and one study examined both. The empirical literature was categorized into three main themes: strategies used to develop a supportive professional relationship, factors influencing the relationship and the nature of these relationships.

**Discussion:**

The results reveal a gap in understanding the nature of supportive professional relationships from the service users’ perspective, as well as how professionals construct these relationships.

## Introduction

The most common definition describes crisis as a subjective experience that threatens and overwhelms a person’s ability to deal with a specific situation using their normal problem-solving abilities, coping mechanisms, or current resources [1, 2]. A crisis therefore can have detrimental effects on mental health (e. g. panic), which also can bear implications on physical health (e. g. headaches). The event of a crisis can lead to long-term harm, e. g. post-traumatic stress disorder. Nizum [2] therefore states that the harmful short-term and potential long-lasting effects of a crisis call for timely and effective interventions.

For many years, psychiatric inpatient care has been the standard care modality for people experiencing a crisis in Germany [3]. More recently, outpatient care continues to become more important not only in Germany but many other countries [4–6]. This is due to possible benefits of community-based care as an alternative to institutionalization [7, 8]. As a result, the World Health Organization identified in their Mental Health Action Plan 2013–2020 “to provide comprehensive, integrated and responsive mental health and social care services in community-based settings” [9] as one of four major objectives, declaring outpatient support a global priority.

Worldwide various services provide outpatient crisis support, such as crisis management services, crisis assessment and treatment services or crisis resolution home treatment teams [10–12]. Considerable differences among these services exist in terms of what they offer (telephone services and/or mobile crisis teams providing home treatment), their accessibility (24/7 or defined opening hours), the cooperation with other institutions (hospitals, fire department, etc.), how clients receive support (no referral/referral through other services), and the intervention models they use [5, 6, 13, 14]. However, they all define crisis intervention as a short-term, low threshold, time-limited, outpatient, and voluntary intervention to cope with crisis [15]. Crisis intervention is a multidisciplinary field which includes social work, psychology, nursing, medicine and many more [8].

An important role in the successful management of a crisis is played by the professional relationship, also referred to as working alliance/relationship, therapeutic alliance/relationship and professional relationship [16]. All these terms refer to the relationship between people experiencing a crisis and professionals working in crisis intervention services helping to overcome the crisis. The most common definition defines it as the agreement on the tasks and goals of therapy and the quality of the relational bond between client and therapist [17].

This relationship has been researched by all the disciplines involved in the management of crises: In social work, the importance of the professional relationship is unquestioned and is considered being the core component of any social work intervention [18]. In psychotherapy, it is “one of the most commonly studied psychotherapy constructs” [19] and is believed to affect every type of therapy [16, 20]. Priebe & Mccabe [21] even argue that the relationship could be therapy in itself. In nursing, it is considered to be an essential component of mental health nursing and a key aspect of the nursing role [22, 23]. In medicine, the professional relationship is seen as an important factor in the effectiveness of treatment [24]. Therefore, evidence on the great influence of professional relationships on mental and physical health is well established. This evidence is transferred into models on how to build a professional relationship and how a good professional relationship should look like [25–27]. Furthermore, there are instruments on how to measure the quality of professional relationships [24].

The existing evidence on professional relationships cannot necessarily be transferred to outpatient crisis interventions because the professional relationship differs depending on the context in which it is developed. For example, there is evidence of a negative association between involuntary admission and the quality of the relationship [28, 29]. Furthermore, professional relationships seem to form faster, more empathetically, and more cooperatively in a home-based setting than in an inpatient setting [30]. Two important outpatient services in mental health care are psychotherapy and case management. In contrast to both, crisis intervention is a short and focused treatment aimed at impeding progression and damage situations for patients and others involved [31]. Being in need of emergency treatment, patients in acute psychiatric settings tend to present with higher and/or more acute degree of suffering [32]. Crisis intervention therefore may require a more active approach by professionals, whereas psychotherapy or case management acts in a slower pace [31, 33]. Furthermore, in crisis situations the professionals have typically less time to establish rapport than in community mental health contexts [34].

Given the increasing popularity of outpatient care and the potential importance of the professional relationship during an outpatient crisis intervention, it is critical to understand on how to establish a supportive relationship in this context. Subsequently, especially during an outpatient crisis intervention, own models are needed to be able to establish and maintain a relationship in the shortest possible time [35]. The currently existing models that guide professionals through a crisis intervention and provide steps for dealing with a person in crisis [8, 14, 35] mostly lack of research-based evidence [35, 36] and remain unspecific regarding the relationship. Johnson et al. [6] state that “service design and development should be rooted in evidence”. Therefore, this review aims to explore this lack in research [32, 37] by examining what kind of empirical literature, and thus what kind of evidence exists regarding the nature of supportive professional relationships during outpatient crisis interventions, to map the key concepts of the described relationship, and to point out research gaps. This review therefore seeks to provide information that will help professionals to better support individuals in crisis.

## Materials and methods

Scoping reviews are used to explore the scope of the literature on a topic [38]. According to Colquhoun et al. [39] a scoping review “is a form of knowledge synthesis that addresses an exploratory research question aimed at mapping key concepts, types of evidence, and gaps in research related to a defined area or field by systematically searching, selecting, and synthesizing existing knowledge”. Therefore, scoping reviews provide a map of the existing literature without quality assessment or extensive data synthesis [40]. This scoping review is reported according to PRISMA-ScR [41] (S2 Table). The objectives, inclusion criteria, and methods were specified in advance and documented in a protocol which was published on OSF [42]. The methodology for this scoping review was based on the framework described by Arksey and O’Malley [43] and the related recommendations of Levac et al. [44].

### Research question

This review was guided by the question, “how is a supportive professional relationship during a crisis intervention described in the empirical literature?” Therefore, this review has three aims:

1. To identify types of evidence related to the professional relationship during a crisis intervention, regardless of its methodological quality, and therefore determine the range of existing evidence available on this topic [45].
2. To map the key concepts by identifying key characteristics of relationships, and thus understand the extent of the knowledge regarding the relationship during a crisis intervention [46].
3. To point out research gaps regarding the professional relationship during a crisis intervention [47].

### Search strategy

Since crisis intervention is usually a multidisciplinary field of work [8], the databases of disciplines involved in crisis intervention (social work, psychology, nursing, medicine) were used to search for relevant literature. The selected databases were Social Science Citation Index (via Web of Science), PsycINFO (via EBSCO), CINAHL (via EBSCO), and MEDLINE (via EBSCO). Possible search terms for the elements of a scoping review—population, concept and context (PCC) [45]—were freely associated. The population of the review includes adults in crisis and professionals, the concept refers to the professional relationship, and the context of this review relates to crisis interventions. The generated search terms were tried out in various search engines and catalogues and supplemented with further terms and synonyms using the hits found. Subsequently, terms were discussed with other researchers and again expended. The search terms were used to develop one individual search string for each database, combining the elements of the review (PCC). To generate as many hits as possible, only “concept” and “context” were used for the search. Related search terms were linked with the Boolean operators “AND”. All search terms were searched for in the title and abstract. In addition to the search terms, the database-specific keyword registers (e. g. MESH) were used to identify relevant keywords, which in turn were added to the search string. To further improve the search string, we used the PRESS guidelines, an evidence-based checklist which is intended to guide and improve the peer review of electronic literature search strategies on ourselves [48] (Table 1).

**Table 1:** Example search string.

**CINAHL via EBSCO**
(TI “crisis intervention*” or AB “crisis intervention*” or TI “crisis management*” or AB “crisis management*” or TI “brief intervention*” or AB “brief intervention*” or TI “brief treatment*” or AB “brief treatment*” or TI “psychosocial intervention*” or AB “psychosocial intervention*” or TI “psychological intervention*” or AB “psychological intervention*” or MH “crisis intervention” or MH “nursing interventions” or MH “crisis therapy” or MH “psychosocial intervention” or MH “psychiatric emergencies” or MH “emergency services, psychiatric” or MH “crisis management” or MH “early intervention” or MH “psychotherapy, brief” or MH “social work, psychiatric” or MH “psychological first aid”)
**AND**
(TI “professional relation*” or AB “professional relation*” or TI “doctor patient relation*” or AB “doctor patient relation*” or TI “physician patient relation*” or AB “physician patient relation*” or TI “nurse patient relation*” or AB “nurse patient relation*” or TI “therapeutic alliance*” or AB “therapeutic alliance*” or MH “physician-patient relations” or MH “nurse-patient relations” or MH “therapeutic alliance”)

See also supporting information (S1 Table)

### Eligibility criteria

The review focuses on the empirical literature regarding the relationship. The term “empirical literature” refers to nearly all stages of evidence of the evidence-hierarchy used in evidence-based practice (EBP) including systematic review of RCTs/individual RCT (level 1), systematic review of cohort studies/individual cohort study/outcome research (level 2), systematic review of case control-studies/individual case-control study (level 3), case series (level 4), and expert opinions (level 5). This hierarchy was developed by the Oxford University Centre for Evidence-based Medicine [49] and is meant to help practitioners quickly appraise the quality of research knowledge [50]. Therefore, only articles which are based on level 1–4 of the evidence-hierarchy were included. Text and opinion papers and models (level 5) were excluded. In cases where systematic reviews and meta-analysis did not report directly on the research question but contained studies that promised to be relevant, they were screened to determine whether they contained studies that met the inclusion criteria. In addition, the treatment of people with mental illness has changed significantly over time and the professional relationship has evolved from a paternalistic approach to one that is more collaborative [12, 51]. The Mental Health Action Plan published by the World Health Organization in 2013 declared outpatient crisis support a global priority [9]. To include discussions predating this plan we included literature published in 2007 or later. Due to limited resources for translation, articles published in languages other than English and German were excluded. If access to certain articles wasn’t possible—not even through interlibrary loan or purchase—the authors were contacted. Only if the authors didn’t grant access the articles were excluded.

#### Participants

The review included studies involving professionals offering crisis interventions and people in crisis over the age of 18.

#### Concept

To be included in the review, there had to be a professional face-to-face relationship.

#### Context

The review included studies on crisis interventions, defined as a short-term, low threshold, time-limited, outpatient, and voluntary interventions to cope with crisis [15]. “Short-term” includes all interventions that are applied without waiting periods in an immediate crisis. “Low threshold” means that the help must be accessible to all persons in crisis, no diagnosis necessary. Furthermore, it must be a “time-limited” offer, which means that the offer ends when the crisis is resolved. Evidence suggests that perceived coercion is related to a more negative relationship [52]. Therefore, only voluntary crisis interventions were included. In addition, interventions where contact between professionals and people in crisis predated the crisis were excluded, as it is assumed that the professional relationship chances over time [53]. Furthermore, only outpatient services were included. This refers to services that visit people in crises at home or other locations, or services that people in crises can get help from without the option of staying overnight.

### Screening and quality appraisal

**Fig 1:**
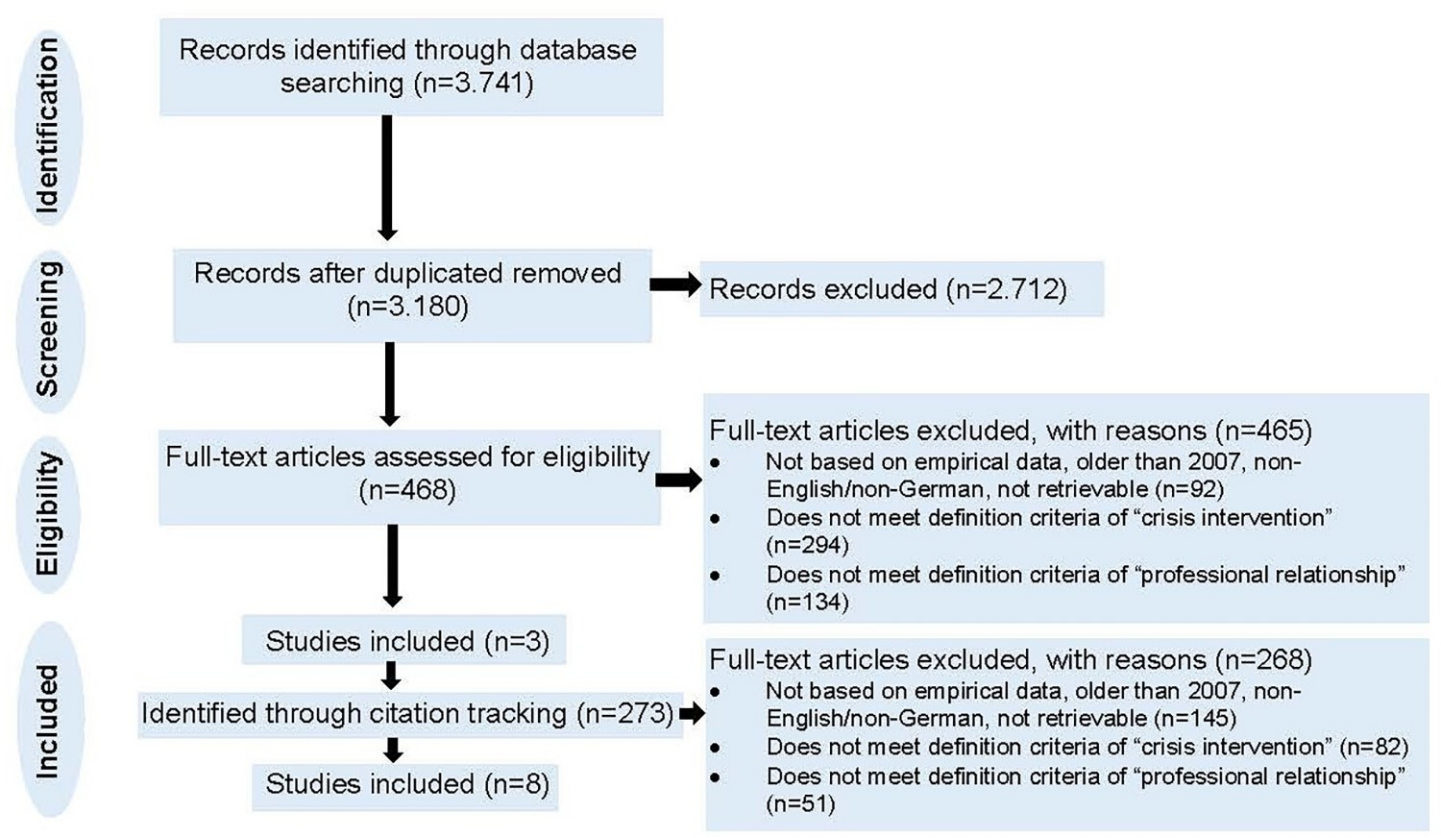
Flowchart.

All citations were imported into the bibliographic manager Zotero. Duplicate citations were removed manually. A two-stage screening process was used, checking all identified citations for relevance using the predefined inclusion criteria. For the first level of screening, titles and abstracts were screened twice by the first author, with all articles where there was uncertainty being reviewed by the third author. Potentially relevant sources were retrieved in full. For the second level of screening the full texts of the remaining citations were assessed in detail by the first author, whereby again uncertain hits were checked by the third author. Reasons for exclusion of full texts are listed in the flow chart (Fig 1). With the remaining full texts, citation tracking using Google Scholar was performed. Therefore, all articles cited in the included full texts were identified (backward searching). In addition, all articles that cited included full texts were also identified and again screened by full text (forward searching).

### Data extraction

A data extraction form was developed and pilot-tested in advance [54] and published in the research protocol. The data extraction was conducted by the first author, although all three authors agreed on the data extraction form published in the protocol. The data extracted contained basic study information (Table 2) and key findings on the professional relationship.

**Table 2:** Characteristics of the included studies.

	aim of the study	setting	method	results
**Giménez-Díez et al. (2020); Spain [57]**	To assess patients’ and their families’ satisfaction	Crisis resolution home treatment team (CRHTT)	Cross-sectional study including quantitative survey data and qualitative interview data (patient n = 20; relatives n = 20)	Patients and relatives reported high satisfaction that seems to be related to person-centered nature of care.
**Giménez-Díez et al. (2022); Spain [55]**	To explore nurses‘ perceptions and constructions about care	Crisis resolution home treatment team (CRHTT)	A qualitative case study using semi-structured interviews with staff (n = 10)	Three main categories emerged from the data analyses: nurses’ perception of the care provided, nursing setting of care at home and nursing care plan at home.
**Hopkins & Niemiec (2007); UK [11]**	To identify what previous users felt was most important to them while receiving the service and to use this information to formulate a service evaluation questionnaire	Home treatment service of the Crisis Assessment and Treatment Service (CATS)	Participatory research methodology used employed a two-stage modified Delphi study and semi-structured interviews (n = 70).	Seven themes emerged from the data: accessibility, availability, consistency, quality, choice/negotiation, communication, changes, and endings.
**Morant et al. (2017); UK [58]**	To investigate service users, providers, developers and carers experiences and views, and what is important in good quality home-based crisis care.	Crisis resolution teams (CRTs)	Semi-structured interviews and focus groups with service users (n = 41), carers (n = 20) and practitioners (CRT staff, managers, and referrers; n = 147, 26 focus groups, 9 interviews) in 10 mental health catchment areas, and with international CRT developers (n = 11).	Three domains salient to views about optimal care were identified: 1. The organization of CRT care, 2. The content of CRT work, and 3. The role of CRTs within the care system.
**O’Reilly (2021); UK [60]**	To elucidate staffs conceptualizations of compassionate care, as well as the perceived barriers to, and facilitators of compassionate care	Crisis resolution teams (CRT)	Individual, semi-structured interviews with staff (n = 12)	Four main themes and several related subthemes were generated from the study data: going the extra mile, the operation of social power, centrality of team processes, and the balancing act.
**Procter et al. (2015); Australia [34]**	To explore the engagement experiences of clinicians to identify the attributes used when engaging with consumers	Community mental health service center	Semi-structured focus groups (n = 2) with 16 clinicians	Two key themes pertaining to the skills and attributes used for successful consumer engagement: 1. Building trust, through communication style, an honest approach, facilitating choice and locating trust networks, 2. Portraying genuine care, through showing respect, offering practical assistance, and taking the least restrictive pathway
**Schwarz et al. (2019); Germany [59]**	To analyse users’ experiences and satisfaction	Counselling service	Semi-structured interviews with service users (n = 9)	Several aspects of the users’ experiences with counselling services have contributed towards their satisfaction with the service. The importance of short waiting times and on-call telephone services as well as sufficient time taken for consultations, the availability of outreach counselling and the quality of the relationship were highlighted. Potential for improvement was seen in the visibility of the service in the community.
**Spiers & Wood (2010); Canada [56]**	To explore the perceptions and actions of community mental health nurses in building a therapeutic alliance in brief therapy and the factors that facilitate or impede its development	Community mental health clinic system	Focus groups with 11 community mental health nurses	Participants described therapeutic alliance as the point at which the clients recognized that the nurse is fully attuned to being in the moment as they connect to their own issues in a positive way. Building an alliance consisted of three nonlinear overlapping phases: establishing mutuality, finding the fit in reciprocal exchange, and activating the power of the client.

### Data synthesis and data charting

According to Pollock et al. [54] “synthesis approaches that aim to reinterpret evidence are not consistent with the purposes of a scoping review”. Rather scoping reviews offer a narrative or descriptive report of the findings [43]. Therefore, this review doesn’t draw conclusions regarding the effectiveness of the relationship due to the absence of a risk of bias assessment or advanced data synthesis. However, to map the key concepts of the described relationship the identified evidence needs to be synthesized. In this case, Pollock et al. [54] recommend the use of a basic qualitative content analyses. Content analysis is a descriptive approach to analysis involving a process of open coding to allocate concepts or characteristics into overall categories, which can be applied to any evidence source or study design in any scoping review [54]. In this scoping review an inductive approach was chosen because “there is a dearth of evidence on the topic” [54]. Therefore, in distinction to the deductive analysis, no predefined framework (e. g. definition of a professional relationship) was applied, but the theory–in this case the nature and therefore definition of a supportive professional relationship during a crisis intervention–emerged during the analysis/extraction process [54]. MAXQDA software was used to perform the analysis. For those articles that focused on a broader research question, such as “satisfaction”, only the results on the relationship were analyzed.

## Results

The first search was conducted in July 2022 in all four databases (Social Science Citation Index, PsycINFO, CINAHL, and MEDLINE). Alerts were set up until the end of March 2023 adding more hits. A total of 3.741 hits were identified. After duplicates were removed, 3.180 studies were reviewed based on their title and abstract using the inclusion criteria, leaving 468 remaining studies for full text analysis. With the remaining 3 [11, 55, 56] studies citation tracking was performed using Google Scholar resulting in an additional 273 hits. After another full-text analysis 5 [34, 57–60] additional studies were identified. A total of 8 studies thus met the inclusion criteria (Fig 1).

### Characteristics of included studies

The included studies describe outpatient crisis interventions in different settings. Four studies [55, 57, 58, 60] focus on crisis resolution teams (CRTs), also known as crisis resolution home treatment teams (CRHTTs), one study [11] focuses on a crisis assessment and treatment service (CAT), another study [34] was conducted at a metropolitan community mental health service center, and one study [59] focuses on a counseling service. One study [56] researched the professional relationship in brief therapy. Even though therapies were normally excluded because of their slower pace, this study was included because it highlights that brief therapy for this study was defined as 10 sessions or less including brief crisis intervention. Therefore, all the included services offered short-term, time-limited, outpatient crisis intervention, which are voluntarily accessed (Table 2).

All of the included studies contained empirical literature according to the evidence-hierarchy by reporting on evidence gained by qualitative interviews [11, 55, 57–60] and/or focus groups [34, 56, 58]. One study included quantitative survey data in addition to the qualitative interviews [57]. However, the quantitative part of the study did not generate any data on the research question, hence only the qualitative data of the study was considered (Table 2).

**Fig 2:**
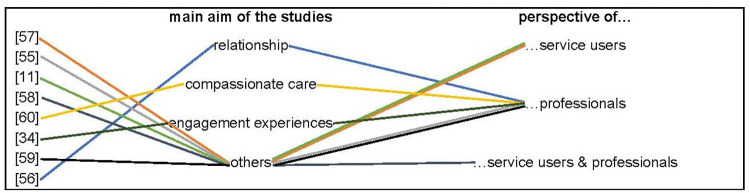
Aim & perspective.

In two studies interviews were conducted with service users [11, 57], and in one study service users and professionals were interviewed [58]. The other five studies focused on the perspective of the professionals [34, 55, 56, 59, 60] (Fig 2).

Only one of the included studies explicitly focused on the relationship, exploring “the perceptions and actions of community mental health nurses in building a therapeutic alliance in the context of brief therapy and the factors that facilitate or impede its development” [56].

Five of the included studies focused mainly on other aspects, while marginally collecting data regarding the relationship (Fig 2). They aimed at investigating the satisfaction [57, 59], at exploring perceptions and constructions about care [55], at identifying what service users felt was most important to them while receiving the service [11], at investigating experience and views of the service [58, 59], and at exploring what is important in good quality home-based care [58]. By focusing on these aspects, they generated data regarding the relationship and where therefore included. For example, one study aimed at assessing patients’ and their families’ satisfaction with care provided by crisis resolution home treatment teams, generating results on the “therapeutic relationship established with the nurse” [57].

One study explored clinicians’ engagement experiences [34]. The broader mental health literature identifies several approaches that are considered essential for successful engagement. Typically, these relate to the attributes and skills of the clinician, with a particular focus on the importance of the professional relationship for clinical outcomes [34]. Thus, the relationship is considered to be a core component of engagement [61]. The included study focused on exploring the views and experiences of clinicians regarding the way they engage with consumers and therefore build rapport [34]. Due to the close association of engagement and professional relationship and the fact that they were used synonymously, the study was included.

One other study focused on elucidating CRT conceptualizations of compassionate care as well as the perceived barriers to, and facilitators of compassionate care within a CRT setting [60]. An essential value in mental health care is the compassion between the therapist and the client, which can strengthen the professional relationship [62]. In the included study “compassion” is used as an attribute of the professional relationship, for example by using the term “compassionate relationship” as a search term prior to the qualitative study [60], which is why the study was included.

Therefore, all the included studies contained evidence on a supportive relationship.

### Description of a supportive professional relationship during a crisis intervention

Of the included studies, the results regarding the relationship were analyzed inductively using qualitative content analysis. For this purpose, codes were assigned which were abstracted into categories. Three main categories were found: “1. Strategies used to develop a supportive professional relationship”, “2. Factors influencing the relationship”, and “3. Nature of a supportive relationship”.

The category “1. Strategies used to develop a supportive professional relationship” contains phrases explicitly describing strategies of professionals on how they positively influence relationship building during a crisis intervention. The second category “2. Factors influencing the relationship” refers to factors that have been described as affecting the relationship but cannot be directly influenced by the professionals. The category “3. Nature of a supportive relationship” contains a description of the result of the process described within the first category.

#### 1. Strategies used to develop a supportive professional relationship

Most of the empirical data of the included studies mentions aspects regarding the development of the relationship. Relationship building is defined as an active reciprocal process of “nonlinear overlapping phases” [56] in which specific strategies are used [11, 56]. These strategies are presented in two subcategories.

*1*.*1*. *…through good communication*. One important factor in the development of the relationship mentioned in the studies is the communication between professionals and service users [11, 34, 55–58, 60], which “enables the therapeutic relationship to become effective” [55]. The most important communicational aspect is reflective and active listening [11, 34, 55, 56, 58, 60]. Other core communication skills reported by the studies are appropriate non-verbal communication [34], affirmation [56], validation [56], normalization [56], and the use of non-technical [11], clear [11, 58], calm [57], respectful [11] and friendly [11] language. Furthermore, it is considered to be essential to adapt communication to each patient [34, 55]. A more controversial strategy mentioned by one study [56] is the use of humor. On the one hand there is a risk of relationship rupture through client misinterpretation, on the other hand humor seems to promote a positive energy flow.

*1*.*2*. *…through actions of the professionals*. In addition to a good communication, behavioral strategies facilitate the development of a relationship, which can be actively used by the professionals [56]. Most codes were assigned to this category. The most important factor mentioned by all the studies is empowerment. Therefore, it is considered important “activating the clients’ power to achieve their goals” [11, 34, 55–60] and equalizing power [55–57] by shared-decision-making [34, 55, 59, 60]. Professionals equalize power by meeting the service users halfway acknowledging that their expertise and effort needs to be matched with the service users available knowledge and energy [56]. Closely linked to the activation of power is fostering hope [11, 55, 56, 58], which is considered to be an intentional relationship building action “related to the capacity for people to see a direction to the achievement of their goals and to feel that they can move in that direction” [56]. The second most important aspects are professionals showing respect [11, 34, 55–58] and offering care which is humanizing, non-judgmental and non-stigmatizing [11, 55–58, 60]. Connecting “human-to-human” [60] and being treated as normal, not like a set of symptoms [11] is therefore considered to be an important engagement strategy. Another important factor is relationship-building through practical support [34, 56–58, 60]. Practical and social interventions are seen as “vehicles for therapeutic relationship formation [58]”. This includes offering assistance beyond their mental health needs such as taking out bins [34, 58, 60], and just being present by visiting regularly [56–58]. Furthermore, building trust [34, 55, 58], being empathetic [55, 57, 59], caring [11, 34, 58], and being friendly [11, 57, 58] are considered to be important. Moreover, important factors mentioned are being reliable [57, 58], honest [34, 55], being helpful and supportive [57, 58], and making sure clients understand [56, 57]. Other factors are taking the least restrictive pathway [34, 55], e. g. in terms of where the interaction takes place, “not being phased by it all” [11], showing sincere interest [57], transmitting confidence and security [57], thoughtful referring [60], and balancing professional and personal disclosure [56].

#### 2. Factors influencing the relationship

Six studies [11, 34, 55, 56, 58, 60] mentioned structures of the services that are considered helpful regarding the development of the relationship. The most often mentioned aspect is having enough time [11, 55, 56, 58, 60], which is closely linked to workload influencing the relationship [56, 60]. Another aspect concerns staff consistency which is mentioned by two studies and is considered to be important for relationship building [58, 60]. However, due to the specific circumstances of crisis services, “it is inevitable that there will be some disruption to the continuity of contact offered to service users” [11]. One other factor influencing the relationship is the willingness and ability of service users and professionals [56, 60] to engage, e. g. because of sympathy [56], personality style [56, 58], and previous experiences and prejudices [56, 60]. Other aspects mentioned to influence the development are legal constraints [56] (e. g. limits of confidentiality [58]), the aim of the intervention, pressure to “discharge”, quantity of quality, and the diversity within the crisis teams [60]. Further aspects concern, driving, weather conditions [56], coercion (insistence of another party to search for treatment) [56], and symptoms of the client [56]. Another study mentions the need for providing the service users with a suitable matched professional [34]. One other study emphasizes the importance of the crisis intervention team, which can help facilitate relationships by empowering staff, help colleagues deal with difficult situations and teach colleagues how to establish relationships [60].

#### 3. Nature of a supportive relationship

Some studies describe the result of the process of developing a professional relationship and therefore, how a supportive professional relationship looks like [11, 55, 56, 58, 59]. The only overarching theme that could be identified over more than one study is that the professional relationship during a crisis intervention is a combination of being like a friend and being like a professional, and therefore containing the “professional and personal self” [11, 56, 59], which is considered being more equal than in acute in-patient wards [56, 58]. One study reports nurses perceiving the relationship “as an intuitively perceived sense of the moment of connection occurring when the client recognized that the nurse was fully attuned and aligned with them” [56]. Therefore, relationship formation results in “a sense of energy alignment toward accomplishment of treatment goals” [56] and could be defined as “bonds of trust” [11].

## Discussion

Scoping reviews “determine the scope or coverage of a body of literature on a given topic and give clear indication of the volume of literature and studies available as well as an overview (broad or detailed) of its focus” [46]. This scoping review shows that only eight studies could be identified that report at least at some point on the professional relationship during a crisis intervention. However, only one of the included studies aims directly at the exploration of the professional relationship. Thus, it is not clear what definition of a relationship underlies the other included studies.

Moreover, those studies that focus on relational aspects concentrate exclusively on the perspective of the professionals, although it can be assumed that relationships always develop reciprocally. There is evidence that the positive influence of a professional relationship is fundamentally determined by the subjective perception of the person in crisis [63]. It has been shown that in the context of mental health, it is not only the actual received support that is relevant, but above all the subjectively perceived support [64].

Furthermore, only one study focuses explicitly on the course of the relationship by identifying phases in which it is developed. However, there is evidence that especially the beginning and ending of the relationship are important for the treatment to be effective [65].

Moreover, the relationship during a crisis is influenced by the context in which it is developed. Examples include the crisis situation itself, capacities to form a relationship, the diagnosis, attachment history, motivation, and symptoms [25, 66]. Some of these influences are reported in the included studies, even though some questions remain. For example, many of the included studies focus exclusively on nursing [55, 56] even though crisis intervention often is conducted in multidisciplinary teams. According to van Haaren et al. [67] in multidisciplinary care the professional relationship becomes more complex and involves different types of relationships.

In summary, although there is a growing focus on the evaluation of outpatient crisis intervention services through recent years [68] research especially for how to build a supportive professional relationship is still lacking. Therefore, it is not possible to really use evidence to guide their relationship building. Murphy et al. [8] state that a major problem “with early community care was that although it could care for people during their relatively stable periods, it was unable to cope with acute phases or relapses”, which is why evidence is needed on how to treat psychiatric crises in the home environment. Professional relationships are sometimes difficult to develop and sustain. Therefore, professionals would benefit from evidence-based support to foster more positive relationships. Ruud & Friis [35] state that “there is a need to adapt professional training in building and maintaining therapeutic relationships to the typical acute care setting, with limited time available and other restrictions”.

In consideration of the evidence found in this review, four questions remain:

1. How must a professional relationship be during a crisis intervention so that it is perceived as particularly supportive from the user’s perspective?
2. How can this supportive relationship be constructed/designed from a professional perspective?
3. How can the course of such a supportive relationship be described?
4. What factors can be identified that influence the relationship?

These questions should be answered with the help of further research to develop evidence-based models for relationship building in crisis intervention and therefore help professionals guide their relationship building.

## Limitations

This scoping review has some limitations. There is the possibility that the review may have missed some relevant studies because articles which didn’t mention the search terms in the title or abstract weren’t included. There may be evidence on the relationship as part of a larger study and research question. Moreover, because we limited the search to “relationship”, “alliance” and “rapport”, studies that focused on parts of the relationship, such as “communication”, may have been missed. Harris et al. [69] for example aim to describe the perceptions of emergency department visits by persons experiencing emotional distress. The results show that the overarching theme that influences patients perception is the communication. The same problem might occur regarding the term crisis intervention, which might not have been used in all relevant articles.

To reduce the potential for selection bias in the identification of relevant literature screening, data extraction, and data synthesis should be done by at least two people [44, 54]. Due to personal resources this was not possible. Therefore, the screening, data extraction, and data synthesis was done by the first author. However, all articles where there was uncertainty were reviewed by the third author and the data extraction form was approved by all three authors.

According to Pollock et al. [54] regarding the basic qualitative content analysis “an inductive or deductive approach will need to be decided upon by the scoping review team during the protocol development stage and subsequently reported within the protocol”. However, because we didn’t know what kind of data to expect we decided on doing a qualitative content analysis after the full text-screening.

Moreover, only German, and English literature was included and there was no critical appraisal of the included studies.

## Conclusions

The existing evidence on professional relationships during a crisis intervention is insufficient to guide relationship building during a crisis intervention. Therefore, this review identified gaps regarding evidence on the professional relationship during a crisis intervention, and thus makes an important contribution to guide further research.

## Supporting information

S1 Table Full search string. (DOCX)

S2 Table PRISMA-ScR-checklist. (DOCX)
