# Supplementary material for: Professional relationships during crisis interventions: A scoping review
Source: PLoS One. 2024 Feb 23;19(2):e0298726. doi: 10.1371/journal.pone.0298726 (PMC10890742; doi:10.1371/journal.pone.0298726)
Supplement: S1 Table — (DOCX) [file pone.0298726.s001.docx]

| **MEDLINE via EBSCO # 1.663**  ( TI “crisis intervention*” or AB “crisis intervention*” or TI “crisis management*” or AB “crisis management*” or TI “brief intervention*” or AB “brief intervention*” or TI “brief treatment*” or AB “brief treatment*” or TI “psychosocial intervention*” or AB “psychosocial intervention*” or TI “psychological intervention*” or AB “psychological intervention*” or MH “crisis intervention” or MH “early medical intervention” or MH “social work, psychiatric” or MH “emergency services, psychiatric” or MH “psychotherapy, brief” )  **AND**  ( TI “professional relation*” or AB “professional relation*” or TI “doctor patient relation*” or AB “doctor patient relation*” or TI “physician patient relation*” or AB “physician patient relation*” or TI “nurse patient relation*” or AB “nurse patient relation*” or TI “therapeutic alliance*” or AB “therapeutic alliance*” or MH “professional-patient relations” or MH “nurse-patient relations” or MH “physician-patient relations” or MH “therapeutic alliance” ) |
| --- |
| **CINAHL via EBSCO # 1.070**  ( TI “crisis intervention*” or AB “crisis intervention*” or TI “crisis management*” or AB “crisis management*” or TI “brief intervention*” or AB “brief intervention*” or TI “brief treatment*” or AB “brief treatment*” or TI “psychosocial intervention*” or AB “psychosocial intervention*” or TI “psychological intervention*” or AB “psychological intervention*” or MH “crisis intervention” or MH “nursing interventions” or MH “crisis therapy” or MH “psychosocial intervention” or MH “psychiatric emergencies” or MH “emergency services, psychiatric” or MH “crisis management” or MH “early intervention” or MH “psychotherapy, brief” or MH “social work, psychiatric” or MH “psychological first aid” )  **AND**  ( TI “professional relation*” or AB “professional relation*” or TI “doctor patient relation*” or AB “doctor patient relation*” or TI “physician patient relation*” or AB “physician patient relation*” or TI “nurse patient relation*” or AB “nurse patient relation*” or TI “therapeutic alliance*” or AB “therapeutic alliance*” or MH “physician-patient relations” or MH “nurse-patient relations” or MH “therapeutic alliance” ) |
| **PsycINFO via EBSCO # 776**  ( TI “crisis intervention*” or AB “crisis intervention*” or TI “crisis management*” or AB “crisis management*” or TI “brief intervention*” or AB “brief intervention*” or TI “brief treatment*” or AB “brief treatment*” or TI “psychosocial intervention*” or AB “psychosocial intervention*” or TI “psychological intervention*” or AB “psychological intervention*” or DE “crisis intervention” or DE “crisis intervention services” or DE “debriefing (psychological)” or DE “psychological first aid” or DE “preventive mental health services” or DE “brief psychotherapy” or DE “psychiatric social workers” or DE “psychiatric hospital staff” or DE “psychiatric nurses” or DE “psychiatric patients” or DE “brief intervention” or DE “psychosocial rehabilitation” )  **AND**  ( TI “professional relation*” or AB “professional relation*” or TI “doctor patient relation*” or AB “doctor patient relation*” or TI “physician patient relation*” or AB “physician patient relation*” or TI “nurse patient relation*” or AB “nurse patient relation*” or TI “therapeutic alliance*” or AB “therapeutic alliance*” or DE “therapeutic alliance” ) |
| **Social Science Citation Index # 232**  TS=( “crisis intervention*” or “crisis management*” or “brief intervention*” or “brief treatment*” or “psychosocial intervention*” or “psychological intervention*” or “psychological first aid” or “brief psychotherapy” or “nursing intervention*” or “psychiatric emergenc*” )  **AND**  TS=( “professional relation*” or “doctor patient relation*” or “professional patient relation*” or “physician patient relation*” or “nurse patient relation*” or “therapeutic alliance” ) |

**S1 Table. Full search string.**
