# Supplementary figures and images for: Professional relationships during crisis interventions: A scoping review
Source: PLoS One. 2024 Feb 23;19(2):e0298726. doi: 10.1371/journal.pone.0298726 (PMC10890742; doi:10.1371/journal.pone.0298726)

| 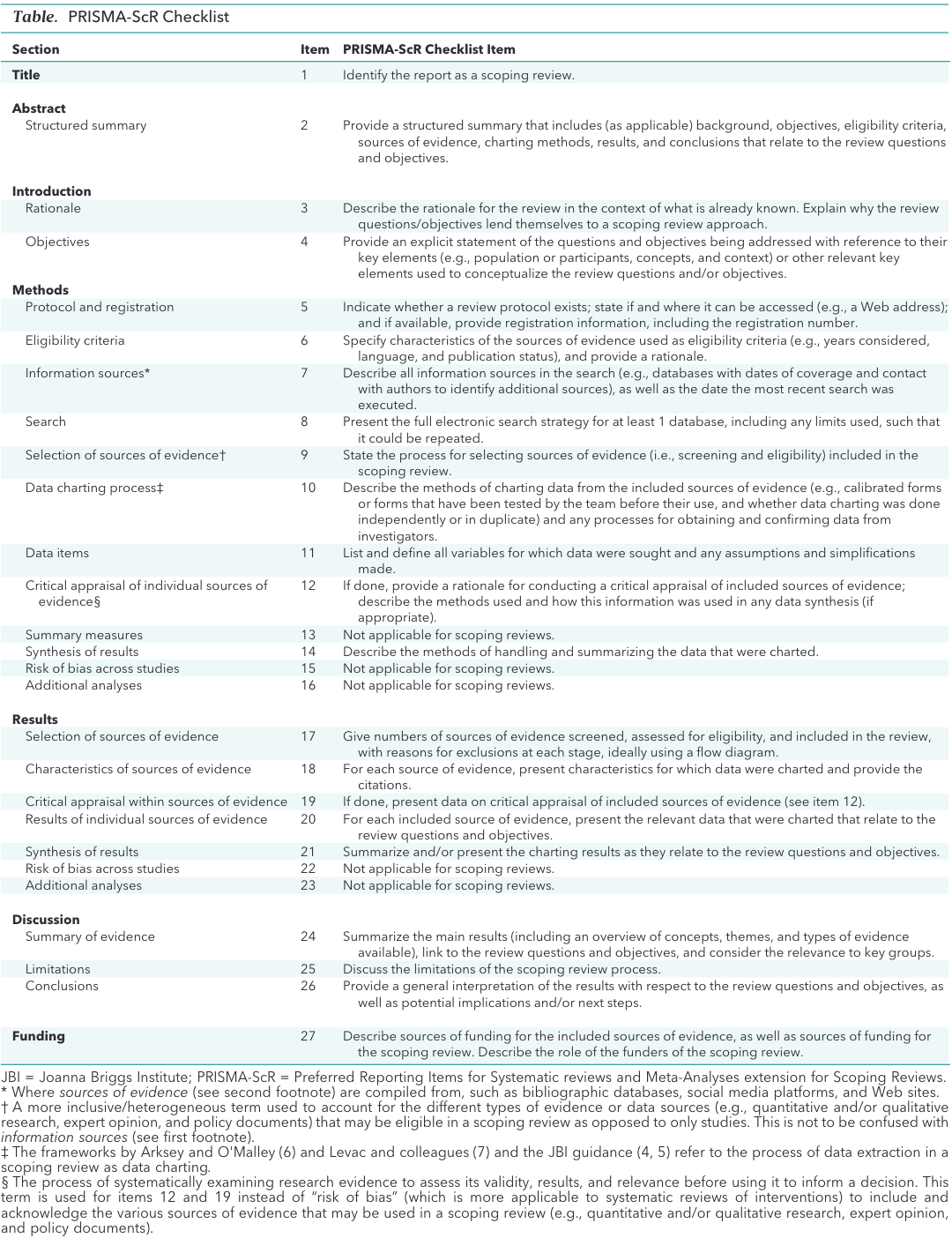 | Reported on page #  1  1-2  2-5  5-6  5  7-8  6-10  7  6-9  9-10  6-9  X  10  9  12-13  X  12-13  11-19  19-20  21  22  22 |
| --- | --- |

**S2 Table. PRISMA-ScR-Checklist. [41]**

Supplement: S2 Table — (DOCX) [file pone.0298726.s002.docx]
